# Seasonal Differences in Effects of Microbial Communities on Duckweed Traits

**DOI:** 10.1002/ece3.72211

**Published:** 2025-09-25

**Authors:** Emma Kinerson, Alex Trott, Anna M. O'Brien

**Affiliations:** ^1^ Department of Molecular, Cellular, and Biomedical Sciences University of New Hampshire Durham New Hampshire USA

**Keywords:** microbiome, phenology, phenotype, phyllosphere, plant–microbe interactions

## Abstract

Host‐associated microbiomes shape the expression of a diverse suite of host traits, ranging from longevity in animals to flowering time in plants. Despite their importance to host traits, the composition of host‐associated microbiomes can fluctuate, and microbiomes of differing composition often have differing impacts on hosts. Fluctuations in microbiome composition can include seasonal shifts that correlate with seasonal phenotypic changes in hosts. Yet, identifying whether these correlated seasonal changes in hosts and microbial communities are causally linked remains difficult because it requires separating the effects of the abiotic environment and host or tissue age from the effects of seasonally altered microbial communities. Here, we used microcosm experiments simultaneously reinoculating axenic duckweeds with microbial communities cultured from different seasons to compare their effects on duckweed growth and trait expression. We found seasonal differences in the effects microbial communities had on duckweeds. Microbial communities cultured from duckweeds in the summer shifted duckweed fronds to be more aggregated (by 5.7%) and slightly more green (by 0.7%) than microbial communities cultured from duckweeds in other seasons, and nonsignificantly increased duckweed growth rates. Inoculation with microbial communities cultured from summer collections also resulted in more total microbial growth in microcosms. These findings enhance our understanding of the influence of seasonality on the complex relationship between microbes and their hosts, with potential future applications in agriculture and medicine.

## Graphical abstract

The microbes living on larger host organisms can have dramatic impacts on their traits. These microbiomes often shift across seasons, mirroring phenological changes in their hosts' phenotypes. In a microbiome‐manipulation experiment, we found that microbes collected during the summer affected duckweed phenotypes differently than microbes collected in other seasons, suggesting that the seasonal shifts in microbiome composition affect host phenotypes.

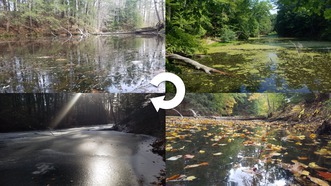

## 1 Introduction

Macroorganisms are deeply affected by the microbiomes living on them, including in their expression of traits as diverse as radiation resistance, body fat, and lifespan in animals (Gould et al. 2018; Guo et al. 2020; Turnbaugh et al. 2008), and flowering time, herbivore resistance, and climate tolerance in plants (Allsup et al. 2023; Howard et al. 2020; O'Brien, Ginnan, et al. 2021). The microbiome that assembles on a host is correlated with host evolutionary and genetic relationships at phylogenetic and genetic timescales (Bouffaud et al. 2014; Fitzpatrick et al. 2018; Grieneisen et al. 2021; Pankey et al. 2022) and can also be characterized as having a “core” make‐up within a species (Stopnisek and Shade 2021). Yet, host‐associated microbiomes are not fixed, and experience major fluctuations in response to environmental and seasonal changes (Björk et al. 2022; Grady et al. 2019; Trivedi et al. 2022). Shifts in microbiomes across environmental changes can feed back to affect hosts (Trivedi et al. 2022), and seasonal cycles in host‐associated microbiomes lead to seasonal changes in the functional repertoire of these microbiomes (Howe et al. 2023), which may have consequences for host traits.

As sessile organisms, plants are particularly exposed to seasonal fluctuations. Understanding seasonal effects on plant‐associated microbiomes is critical because they have outsized impacts on plant nutrient uptake, stress tolerance, pathogen resistance, and myriad other traits, including all aspects of phenology (Friesen et al. 2011; Lindow and Leveau 2002; O'Brien, Yu, et al. 2020; Pieterse et al. 2014; Ramírez‐Flores et al. 2017). Indeed, plant‐associated microbiomes are central to agricultural productivity, ecosystem function, and biodiversity preservation (Mishra et al. 2020; Peixoto et al. 2022; Prasun et al. 2020). As in other host systems, the composition and effects of plant‐associated microbiomes can shift across environmental factors, such as temperature and precipitation (Ruan et al. 2023; Santoyo et al. 2021), and may also be driven by seasonal environmental change (Grady et al. 2019; Howe et al. 2023).

Because plants often reach peak growth rates in spring or summer and are dormant in winter, it is unsurprising that plant phenology and plant microbiome composition shift in tandem across seasons. Yet, the nature of this relationship is unclear: plants could use microbiomes as cues for germination, growth, or reproduction (Chialva et al. 2016; Guimarães et al. 2013), changes in plants could cue changes in microbes (Gianinazzi‐Pearson et al. 1989; Zhao et al. 2021), or plants and microbes could independently respond to seasonally changing abiotic factors (Dombrowski et al. 2017). Because environment, microbiome, and plant phenology or state change all at once during the seasons, identifying causality between these factors is difficult to impossible in field settings (Dombrowski et al. 2017; Ginnan et al. 2022). We identified duckweeds as a study system particularly tractable for disentangling these factors in a laboratory common garden experiment.

Due to their rapid generation time, small size, and existing axenic protocols (Laurich 2019; Thingujam et al. 2024), duckweeds ( *Lemna minor* , *sensu lato*) offer unique experimental opportunities. Duckweeds are also an important aquatic plant, with applications in bioremediation, bio‐fertilizers, animal feed, biofuels, and more (Acosta et al. 2021; Thingujam et al. 2024). Here, we leveraged the experimental tractability of duckweeds to identify fitness impacts of microbial communities collected from different seasons in a single common garden experiment conducted in a growth chamber. Because duckweeds reach their fastest growth rates during the summer (Debusk et al. 1981; Rejmánková 1973), we predicted that plants inoculated with duckweed‐associated microbes collected during the summer would grow faster than duckweeds inoculated with duckweed‐associated microbes collected during other seasons. Similarly, because microbial growth in slow‐moving freshwater, the habitat of duckweeds, also peaks during the summer (Jones 1970; Kirschner and Velimirov 1997; Woodhouse et al. 2016), we likewise predicted that microbes collected from duckweeds during the summer would grow faster than microbes collected during any of the other seasons.

## 2 Methods

We performed a “common garden” experiment to inoculate axenic duckweeds with microbes collected from their home site, comparing the effects of microbes collected from several different seasons in one synchronous experiment in a growth chamber.

### 2.1 Duckweed Lines

To culture isogenic duckweed lines from two collection sites in Durham, NH (Thompson Farm and LaRoche Pond, Table A1), we first collected fronds from the field at each of these sites into a 50 mL centrifuge tube on May 7 (Thompson Farm) and May 12 (LaRoche Pond) of 2022. We identified that these duckweeds belonged to *Lemna minor* , *L. turionifera* , or a hybrid of the two, based on a dichotomous key of morphological features designed for the New England region of the United States (Native Plant Trust n.d.). Phenotypic resolution to species is not possible without molecular diagnostics, though plants isolated in eastern North America are often hybrids (Braglia et al. 2021; Ernst et al. 2025) (M. Frederickson, personal communication). We placed a single frond from each site into a 24‐well plate and allowed it to vegetatively reproduce until it filled the plate. Our duckweeds are likely to be exclusively asexual: duckweeds are expected to have low segregating diversity within an individual parent frond even if they were able to sexually reproduce (Ho 2018), and our duckweeds were never observed to flower. Indeed, triploid hybrids between *L. minor sensu stricto* and *L. turionifera* lack the ability to flower (Ernst et al. 2025; Michael et al. 2025). We therefore consider these single‐frond descent lines to be isogenic, or nearly isogenic.

We then created axenic cultures from each isogenic line following an existing protocol (Laurich 2019). Briefly, we vortexed duckweeds in phosphate‐buffered saline to loosen external microbes, then submerged them in 1% bleach solution for 1 min and 30 s. Following the bleach submersion, we rinsed the plants three times with sterile water. The first rinse lasted 44 s, with the following two rinses lasting 10 min each. We then placed these surface‐sterilized duckweeds into sterile Krazčič media (K media) (Krazčič et al. 1995) and allowed them to grow. We tested for the presence of any remaining microbes able to grow externally to the frond by placing individual duckweeds from each new putatively axenic culture into sterile K media enriched with yeast and mannitol.

### 2.2 Microbe Collection and Inoculum

We sampled duckweed microbiomes from the same two locations as the duckweeds across all four seasons (seven timepoints from La Roche Pond, eight from Thompson Farm, Table A2). We collected samples of duckweeds and associated water into sterile 50 mL centrifuge tubes at the edge of a body of water using sterilized metal spatulas and gloves rinsed with 70% ethanol. We rinsed collection tubes in the body of water three times before collecting duckweeds and microbes. In a biosafety cabinet, we used a sterile inoculation loop to pulverize a single duckweed frond cluster from the 50 mL tube on the inside cover of a petri plate that contained sterile yeast mannitol agar media. We streaked a dab of the pulverized tissue onto the plate media, incubated at 30°C until colony growth covered approximately half the plate area (~5 days), and then stored the plates at 4°C until use. Storage at a common temperature may have eroded some of the compositional differences that occur in microbiomes across seasons as microbes differentially survive at 4°C (Stinson et al. 2022). Indeed, even glycerol preservation can decrease the differences in plant impacts relative to differences observed in original microbiomes (Panke‐Buisse et al. 2017). Yet, storage was necessary in order to run a common garden experiment with microbes collected at different timepoints from the field.

To create the experimental inocula from the plated microbe samples, we swabbed stored petri plates (15 inocula, one for each site and date) using a sterile inoculation loop and stirred them into 15 mL culture tubes containing sterile yeast mannitol media. We cultured the inocula for 2 days in a shaking incubator at 30°C and 300 RPM. Using a BioTek Cytation 5 Plate Reader, we measured optical density (OD) at 450 nm, and OD 600 nm to estimate cell concentration (O'Brien, Laurich, et al. 2020). OD600 provides a standard measure for cell density. OD450 relates to cell density, but is more sensitive to pigments and secondary metabolites, potentially providing an uncorrelated measure if microalgae respond differently to treatments than most microbes (Beal et al. 2020; Di Caprio 2020). We then diluted all inocula to approximately 5,000 cells/μL in sterile Hogland's media.

### 2.3 Experiment Design and Set Up

We separately inoculated each axenic isogenic line of duckweeds (2) with microbial communities from each date of collection from their home site (seven or eight different experimental inocula) or left them uninoculated (one additional treatment for each duckweed line) in a randomized block design in 96‐well plates (four blocks). In each block, we randomly assigned each of the seven or eight inocula treatments for each duckweed line to a full column of a plate, with all treatments for a duckweed line grouped adjacently. This generated eight within‐block replicates (eight wells per column), and each treatment occupied a total of 32 wells across all blocks (see Table A2 for detailed layout).

We prepped well plates by filling wells with 600 μL of sterilized Hoagland's media, then placing axenic duckweed fronds in each well. We then sealed plates with a cell‐barrier seal (BreathEasier, Diversified Biotech). For each microbial inoculum, we delivered 10 μL directly to wells indicated in the randomized block design by puncturing through the BreathEasier seal with the pipette tip. Once all wells were inoculated, we removed the BreathEasier seal and replaced it with a new cell‐barrier seal (BreathEasy, Diversified Biotech) to prevent cross contamination. Finally, we placed plates into a growth chamber for 12 days (photoperiod 17 h light at 23°C and 7 h dark at 18°C).

### 2.4 Measurements

We imaged all experimental plates on day 0 and day 12 from below using an iPhone 13 cell phone camera and a plexiglass table within the growth chamber. After imaging, we measured the OD of the media at two different wavelengths (450 nm, OD450, and 600 nm, OD600) to estimate the density of microbes. We excluded a few datapoints measuring an OD ≥ 1, which occurred only when duckweeds blocked the optical path.

We used ImageJ (Schneider et al. 2012) to measure duckweed growth in square millimeters (converted from pixel area using plate width in images), and to record duckweed traits. We used thresholds of saturation (minimum 89), hue (maximum 121), brightness (maximum 144 or 190, depending on whether a shadow was present) to identify duckweed particles at a minimum particle size of 300 pixels. For all duckweed particles in a well, we averaged the color intensity (0–255 scale converted to 0%–100%), and percentage of split‐channel RGB values in the green channel. We also took a ratio between the total area and total perimeter of duckweed particles, which we call “frond aggregation,” and which measures the tendency of mother fronds to retain daughter fronds without abscission. Plant tissue color is linked to chlorophyll content and stress (Adamsen et al. 1999; Keenan et al. 2014; Negrão et al. 2017), frond aggregation may respond to stress in duckweeds (Henke et al. 2011; Newton 1977; O'Brien, Yu, et al. 2020), and other previous work on duckweeds links variation in these traits to inoculation with different microbes (O'Brien, Laurich, et al. 2020; O'Brien, Laurich, and Frederickson 2024).

### 2.5 Statistical Analysis

**FIGURE A1:**
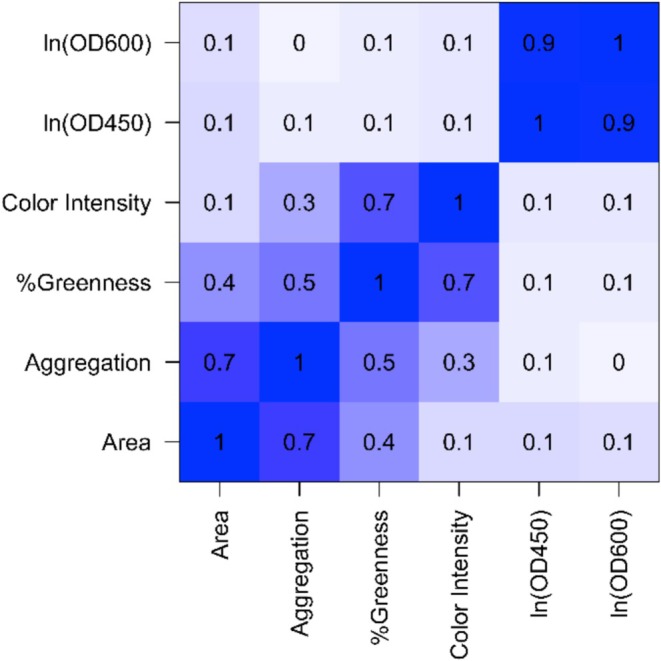
Correlation values of all measured response variables with one another. Correlations in this figure include all the non‐NA values for all traits across all treatments (including uninoculated treatments). Cell background color reflects the strength of the correlation from a correlation of 0 (white) to 1 (blue).

We conducted statistical analyses with R (version 4.3.3) (R Core Team 2024), fitting all linear models with package MCMCglmm (Hadfield 2010). Before analyses, we checked the normality of the data with a Shapiro–Wilk test. Duckweed response variables (frond area, greenness, color intensity, and aggregation) were all approximately normally distributed (0.90 < *W* < 0.99). OD measures were not normally distributed (*W* = 0.68 and *W* = 0.51 for 450 and 600 nm, respectively), but taking the natural log improved normality (*W* = 0.86 and *W* = 0.78 for 450 and 600 nm, respectively). All subsequent analyses therefore used log‐transformed OD measures. Although we analyzed each response variable separately below, some duckweed traits were co‐correlated. The two OD measures were co‐correlated, and these therefore do not necessarily represent fully independent responses to treatments (Figure A1).

To evaluate the effect of inoculating microcosms with any microbes compared to not inoculating with microbes, we fit linear models of the form $\text{Response}\sim \alpha +\beta_{I}*\text{Inoculation}+N_{\text{Plant}}\left(\epsilon ,0\right)+N_{\mathrm{Rep}}\left(\epsilon ,0\right)$, for each response variable. In the model, α indicates the intercept; β indicates the fitted effect of inoculating microcosms versus leaving them uninoculated. The additional error distributions represent a random effect of isogenic line to account for differences in starting fronds across the two lines, and a random effect for differences across groups (eight levels, one group from each block for each isogenic line) to account for spatial effects. We fit this model for each response variable and evaluated significance using pMCMC values (“*p*‐values,” hereafter). Our hypotheses expected that microbial communities from summer collections would have a different effect on duckweeds than microbial communities collected during other seasons. We therefore used a second linear model structure to evaluate the effect of inoculating with microbes cultured from summer collections (June, July, and August; three treatments for each isogenic line, Table A2) versus inoculating with microbes cultured from collections during any other season combined (May, September, October, November, December; four or five treatments for each isogenic line). We excluded data from uninoculated wells for this analysis. For each response variable we fit the model: $\text{Response}\sim \alpha +\beta_{S}*\text{Summer}+N_{\text{Plant}}\left(\epsilon ,0\right)+N_{\mathrm{Rep}}\left(\epsilon ,0\right)$. Here, β indicates the fitted effect of inoculating with microbial communities cultured from summer duckweed microbiomes relative to any other inoculated microbial community; all other terms remain identical to the model used to test the effect of inoculation.

Finally, we also explored the contribution of the two types of treatment (the source site for plants and microbes, and each unique microbial collection event) to explain variation in the measured variables. To do this, we divided the treatment sums of squares for the source site of the duckweeds (summed squared distances of each datapoint to its treatment mean) by the total sums of squares (summed squared distances of each datapoint to the grand mean). This was the portion of the variation explained by the source site (for both duckweed genotype and microbial communities). Next, we calculated the treatment sums of squares for each unique microbial treatment, subtracted the treatment sums of squares for the source site (since the effect of the source site is fully contained in the unique microbial treatments), and divided by the total sums of squares. This was the portion of the variation explained by the unique microbial collection events. We multiplied proportions by 100 to yield percentages of variation explained (O'Brien, Laurich, and Frederickson 2024).

## 3 Results

We aimed to understand the effects of microbial communities collected from summer or other seasons on duckweed phenotypes and fitness. Using a “common garden” experiment within a growth chamber, we compared the effects of microbes collected from several different seasons from the home source site for two duckweed lines in one synchronous experiment.

**FIGURE 1:**
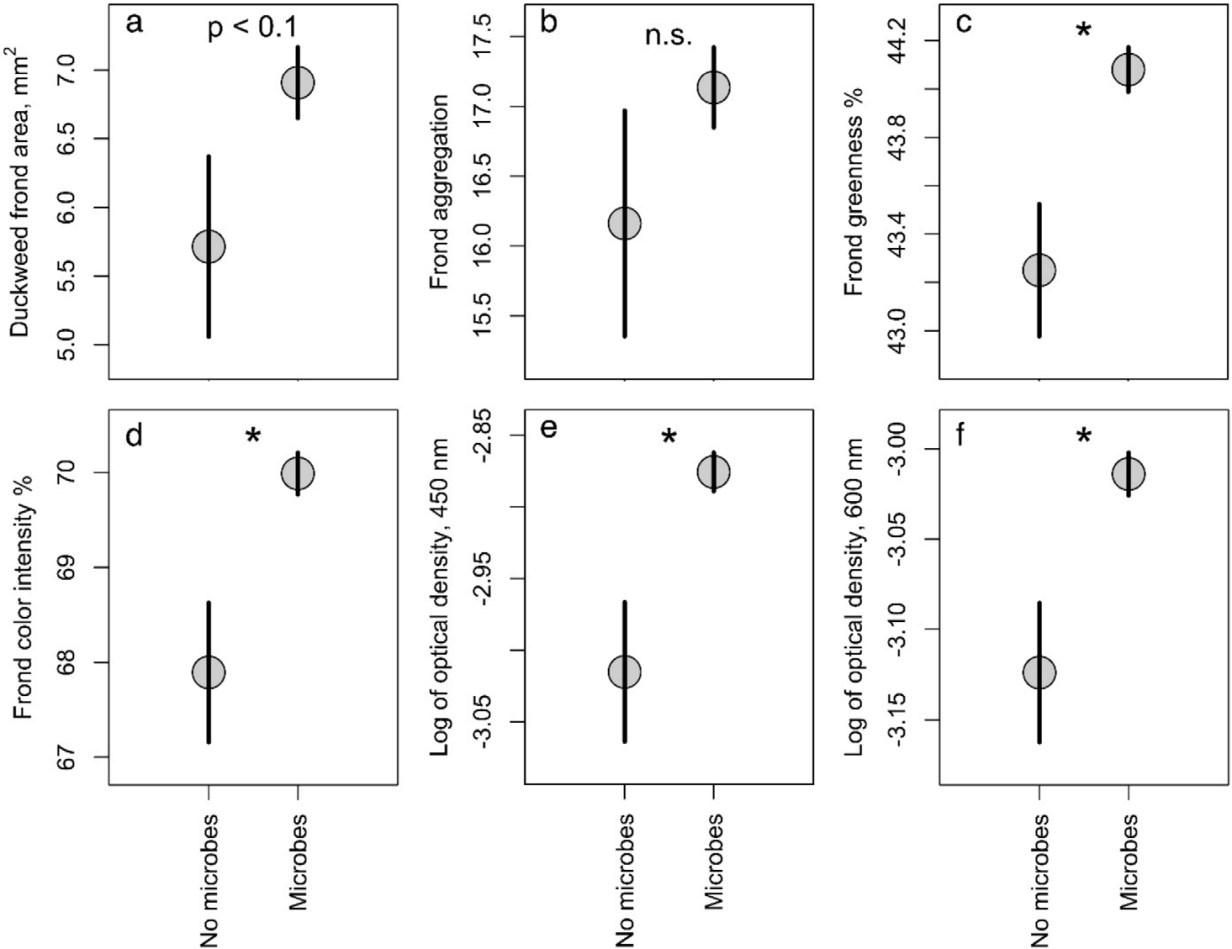
Effects of microbial inoculation on measured response variables—(a) final duckweed frond area, (b) duckweed frond aggregation, (c) frond greenness, (d) frond color intensity, and OD of final media at 450 nm (e) and 600 nm (f). Points show the treatment means, with bars representing plus or minus one standard error of the mean. Asterisks indicate significant differences (*p* < 0.05) between treatments. Both OD measures, at 450 and 600 nm, were transformed by the natural log before analysis; see Section 2.

Duckweed microcosms inoculated with any microbes differed in most measured responses from uninoculated microcosms (Figure 1). After 12 days, inoculated microcosms yielded duckweeds with significantly higher frond greenness (2%) and color intensity (3.6%) (βs for inoculation effect both positive at *p* < 0.05), and marginally higher frond area (9.8%) (*p* < 0.1), but no difference in frond aggregation when compared to uninoculated microcosms (*p* > 0.1, Figure 1a–d). In other words, the presence of microbes significantly changed duckweed frond color and was consistent with a marginal increase in duckweed fitness in this experiment.

**FIGURE A2:**
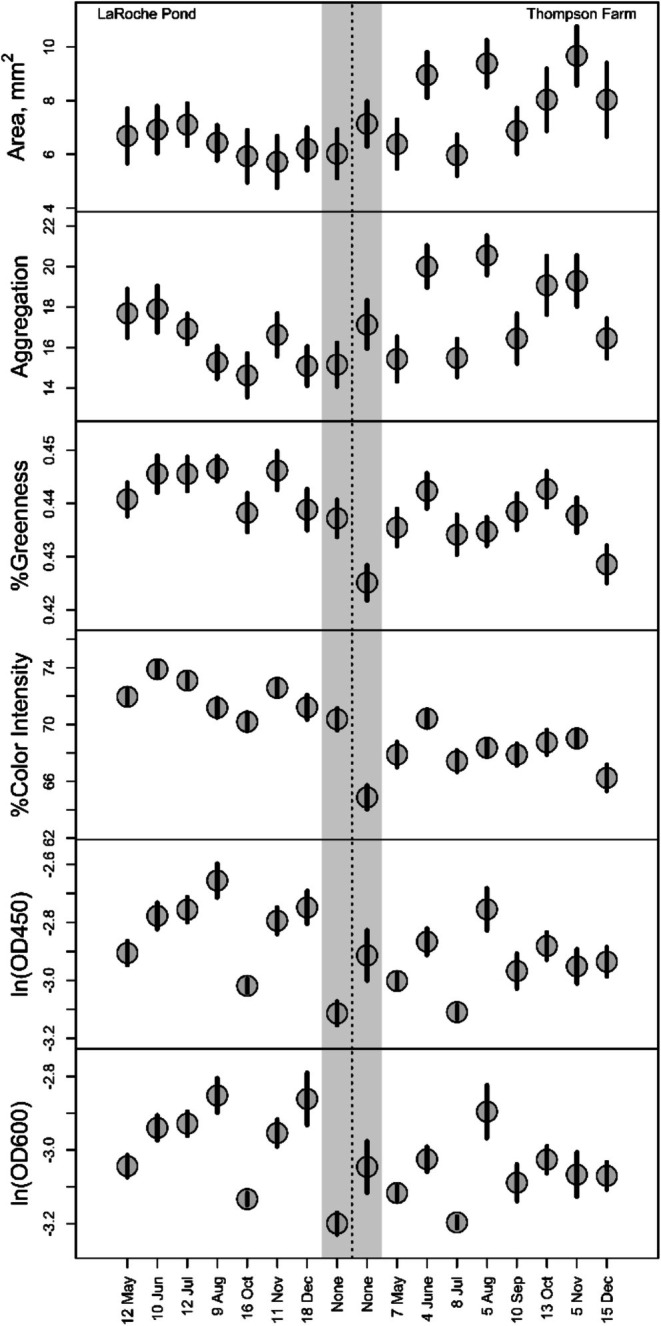
Means (points) and standard errors of the mean (bars, plus or minus one standard error from the mean) for each measured response in each treatment. Data for duckweeds and microbes collected from LaRoche Pond are on the right of each panel, whereas data for duckweeds and microbes collected from Thompson Farm are on the left of each panel. Points on the gray background are treatments in which no microbes were inoculated.

Inoculated microcosms also yielded significantly higher OD measures, compared to uninoculated microcosms, with increases of approximately 3%–5% at both 450 and 600 nm (*p* < 0.05, both wavelengths, on a log scale). This is expected given that microbial cells are the primary contribution to OD measures, and uninoculated microcosms were only expected to have microbes if they were contaminated during setup. Indeed, OD at 600 nm is a good proxy for the density of microbial cells in duckweed microcosms (O'Brien, Laurich, et al. 2020). However, in microcosms with Thompson Farm duckweeds, a few uninoculated wells had high OD values (three adjacent wells in Plate 1 have OD600 > 0.1), raising the average OD of uninoculated wells (Figure A2). We anticipate that this represents a contamination event during the experiment setup, though we did not exclude these data from analyses. Contamination, or simply the difference in sample size, may explain the relatively higher standard error in inoculated treatments (Figure 1), and potentially the lack of significance in some contrasts.

**FIGURE 2:**
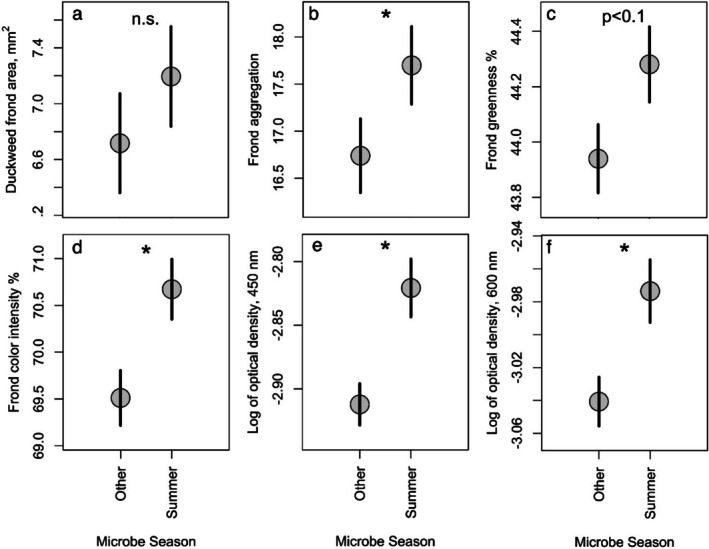
Effects of inoculation with microbial communities cultured from summer microbiomes versus other microbiomes on measured response variables—(a) final duckweed frond area, (b) frond aggregation, (c) frond greenness, (d) frond color intensity, and OD of final media at 450 nm (e) and 600 nm (f). Points show the means for all datapoints from all treatments within the respective category (microbes from summer or not), with bars representing plus or minus one standard error of the mean. Asterisks indicate significant differences (*p* < 0.05) between treatment categories (microbes from summer or not). Both OD measures, at 450 and 600 nm, were transformed by the natural log before analysis; see Section 2.

Microcosms inoculated with microbial communities cultured from summer duckweed microbiomes differed in most measured variables from microcosms inoculated with microbial communities cultured from duckweed microbiomes in any other season (Figure 2). Specifically, microcosms inoculated with microbial communities from summer duckweed microbiomes yielded duckweeds with fronds that were more aggregated (5.7%), greener (0.7%), and had higher color intensity (1.8%) (βs for summer source effect all positive, *p* < 0.05 for aggregation and color intensity, < 0.1 for greenness). They also produced duckweeds with a non‐significant increase in frond area (5.7%). These results suggest that microbes present during the summer may substantially alter the expression of duckweed traits, without substantially impacting fitness. Microcosms inoculated with microbes collected during the summer yielded more total microbial biomass than those inoculated with microbes from other seasons as measured by OD600, and both OD metrics were higher in these microcosms (both *p* < 0.05; 2%–3% higher on a log scale).

**FIGURE 3:**
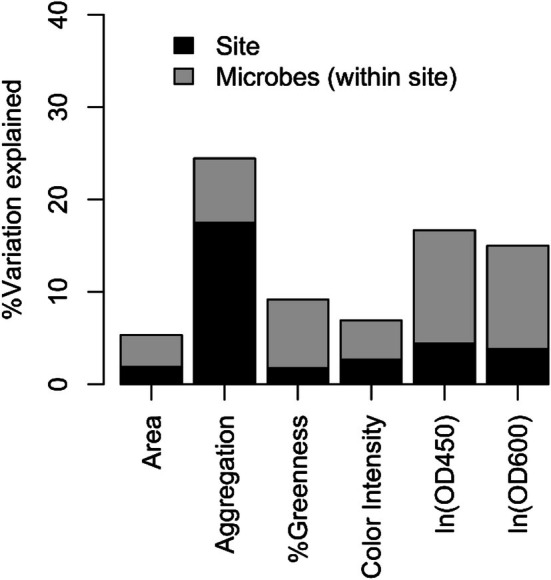
The percentage of the total sums of squares of each measured response variable explained by source site location and by the unique microbial communities applied (treatment sums of squares). Area, aggregation, percent greenness, and color intensity all refer to the size (area) or traits of duckweed fronds. “ln” refers to the natural log: both OD measures, at 450 and 600 nm, were transformed by the natural log before analysis; see Section 2.

The source site of the duckweeds and microbes explained less variation than the unique microbial community collection events for all measured variables except frond aggregation (Figure 3). Specifically, the source site of the duckweeds and microbes explained < 3% of the variation in duckweed frond area, greenness, and color intensity in microcosms (1.7%–2.7%), and less than 5% of the variation in OD measures (3.8% and 4.4% for 450 and 600 nm, respectively). However, the source site had a large effect on duckweed frond aggregation, explaining 17.5% of the variation. The unique microbial community applied within source sites (each collection date at each site) explained 11.2% and 12.3% of the variation in OD at 450 and 600 nm, respectively. The unique microbial community applied also explained 6.9% and 7.4% of the variation in frond aggregation and greenness, but only 3.4% and 4.2% of the variation in frond area and color intensity, respectively.

## 4 Discussion

Host‐associated microbiomes seasonally fluctuate in taxonomic composition (Björk et al. 2022; Grady et al. 2019), resulting in turnover in the abundance of species containing different metabolic functions (Howe et al. 2023). These seasonal microbiome fluctuations often correlate with phenological changes in plants (Ginnan et al. 2022; Shade et al. 2013), suggesting the possibility that seasonal changes in the functional composition of host‐associated microbiomes contribute to phenological change in hosts. Here, we used a microcosm experiment to identify whether the culturable portion of the duckweed microbiome differs in its phenotypic impact on duckweed hosts according to the season in which the microbiome is sampled. Because duckweed species grow fastest in the summer (Debusk et al. 1981; Rejmánková 1973), we predicted that inoculating duckweeds with microbial communities from summer duckweed microbiomes would support more duckweed growth. Instead, we found that microbial communities cultured from summer duckweed microbiomes non‐significantly increased duckweed growth but significantly altered nearly every other measured variable when compared to microbial communities cultured from duckweed microbiomes during other seasons (Figure 2). In particular, inoculation with microbes collected in the summer increased frond greenness and color intensity, changes that may indicate a potential increase in fitness via higher chlorophyll content and photosynthetic capacity, despite the lack of significant growth differences (Adamsen et al. 1999; Keenan et al. 2014; Negrão et al. 2017).

Intriguingly, the inoculation with summer‐collected microbial communities moved duckweed phenotypes in the same direction as inoculating with any microbial community (relative to no microbes, compare Figures 1 and 2), though microbial communities collected at many timepoints induced shifts relative to the uninoculated state (Figure A2), and microbial density was not correlated to frond trait values (Figure A1). Because plant trait expression is often tightly linked to the full spectrum of available macronutrients and micronutrients (Baxter et al. 2008; O'Brien, Sawers, et al. 2024; Ramírez‐Flores et al. 2017), we speculate that duckweed trait expression in our microcosms was limited by nutrients the inoculated microbial communities affected, and that impacts on nutrient availability varied between summer‐collected communities and other communities. Indeed, nitrogen fixation, production of iron siderophores, and phosphorus solubilization occur in microbes naturally associating with duckweeds and in cultured strains known to benefit duckweeds (Duong and Tiedje 1985; Ishizawa et al. 2017; Makino et al. 2022), some of which are also known to be seasonal (Elliott 2010; Murrell and Lores 2004; Woodhouse et al. 2016). Alternatively, duckweed traits might be altered by other biomolecules: in addition to fixing nitrogen, cyanobacteria can produce toxins that influence the expression of duckweed frond traits (Rzodkiewicz and Turcotte 2024).

Microcosms inoculated with microbial communities cultured from summer duckweed microbiomes had more total microbial growth (Figure 2, inferred from OD at 600 nm). Freshwater microbes tend to reach peak growth in the summer (Jones 1970; Kirschner and Velimirov 1997), and microbes collected during the summer may grow faster, especially at warm temperatures (Kritzberg and Bååth 2022), suggesting that there is a greater prevalence of freshwater microbes with fast‐growing traits in the summer. Although we know species composition in microbiomes seasonally changes, plasticity and evolution could also contribute to differences in growth rates between our communities cultured from different seasons. Microbes can respond to environmental conditions with changes in the capacity for growth: they can enter dormancy during “poor” conditions and leave dormancy in “good” conditions (Bradley 2025; Jones and Lennon 2010), with lag phases when switching from nongrowing to growing states that reduce total growth (Bertrand 2019). Perhaps more dramatically, large population sizes and short generation times enable some microbial species to cyclically evolve across seasons (Rohwer et al. 2025), potentially resulting in microbes evolving faster growth rates during the summer.

Our intermediate culturing method exposed all microbial communities to the same temperature conditions for almost one year, likely reducing the influence of plasticity relative to evolved and compositional effects, and probably also eroding the compositional similarity between collected and applied microbiomes (O'Brien, Laurich, and Frederickson 2024). Preserving the microbial communities in glycerol stocks might have minimized these changes (Pascual‐García et al. 2025; Rose and O'Brien 2025), but even glycerol preservation can alter the phenotypic effects of microbiomes on plants. Indeed, in at least one case, media cultivation was more effective than cryopreservation at maintaining a microbiome's effect on host traits (Panke‐Buisse et al. 2017). Still, we expect that the effects we observed here may underestimate the phenotypic consequences of seasonal microbiome change for duckweeds in the field. Going forward, we suggest using freshly collected or experimentally primed microbiomes to best preserve effects.

In previous results from duckweeds and other plants, variation in host genotype usually contributed more substantially to variation in host phenotypes than variation in inoculated microbial communities (O'Brien, Laurich, and Frederickson 2024; O'Brien, Sawers, et al. 2024; Wagner et al. 2014; Wendlandt et al. 2021). Yet, the exact identity of the microbial community we applied explained more variation in our measured response variables than the source site (Figure 3), which includes effects of host genotype and any consistent difference in community composition across sites. This more limited role for host genotype (except for frond aggregation) is somewhat surprising, though it has been observed before (Iriart et al. 2024). However, though our sites are in different watersheds, they are located very close together (~1.5 km, see Table A1), and our duckweed isogenic lines may be more genetically similar to each other than genotypes in these previous studies, artificially deflating the contribution of host genotype.

A subset of the effects of microbial community collection date appears potentially clinal (Figure A2, e.g., frond measures for LaRoche Pond duckweeds), though we did not have sufficient collection dates to test this. Indeed, microbial communities can exhibit turnover at the scale of weeks (Bolaños et al. 2022; Grady et al. 2019), days (Björk et al. 2022), or even hours (Allaband et al. 2024; Hubbard et al. 2023). Some microbial communities may have steep changes over short windows (Björk et al. 2022; Fenn et al. 2023), whereas others may turn over more gradually, potentially matching the rate of change in the environment (Bolaños et al. 2022; Fenn et al. 2023). Effects of seasonally driven microbiome shifts on hosts may therefore have detectable effects at a much finer temporal scale than we considered here, and higher density microbiome sampling may be enlightening. To capture these dynamics, we would suggest pairing a biweekly sampling design with intermittent windows of fine (daily) and ultrafine (hourly) sampling. We further suggest collecting environmental and sequencing data. Season is not a linear progression of date, but of changes in temperature, nutrients, and other environmental parameters (Lv et al. 2014), and while studies of *Lemna* microbiome composition exist (e.g., Acosta et al. 2020, 2023; Inoue et al. 2022; O'Brien, Yu, et al. 2020), sources of variation in microbiome composition are less studied.

Here, we saw that seasonal differences in summer microbiomes resulted in different impacts on trait expression in their duckweed host plant. Understanding if seasonal microbiome effects are common across hosts and identifying how they arise and progress will be key next steps. Even if seasonal differences in the effects of microbiomes are ubiquitous, it may yet be the hosts driving shifts, or microbiomes and hosts may engage in seasonal feedbacks. Indeed, host plants appear to change what microbes they select for in the rhizosphere across seasons (González‐García et al. 2023; Zhao et al. 2021). Going forward, experimental work priming and then testing microbiomes and hosts in different “seasonal” conditions in laboratory experiments could begin to tease out these effects, and molecular work could identify the mechanistic connections between seasonal shifts in microbiomes and impacts on host phenotypes, as in Lu et al. (2018). Finally, detailed sequencing studies could consider whether, like free‐living microbes (Rohwer et al. 2025), host‐associated microbes also cyclically evolve, and evolutionary experiments could consider whether seasonally derived microbiome effects evolve more in host genomes or in microbial genomes (O'Brien, Jack, et al. 2021). This knowledge could feed into practical efforts to design microbial inoculant products for agriculture and medicine, where their use is increasingly popular (Li et al. 2023; Shekarabi et al. 2024).

## Author Contributions

**Emma Kinerson:** formal analysis (equal), investigation (equal), validation (equal), visualization (lead), writing – original draft (lead), writing – review and editing (equal). **Alex Trott:** conceptualization (equal), data curation (lead), formal analysis (supporting), investigation (equal), methodology (equal), writing – original draft (equal). **Anna M. O'Brien:** conceptualization (equal), data curation (supporting), formal analysis (equal), funding acquisition (lead), investigation (equal), methodology (equal), project administration (lead), resources (lead), supervision (lead), validation (equal), visualization (equal), writing – original draft (supporting), writing – review and editing (equal).

## Conflicts of Interest

The authors declare no conflicts of interest.

## Data Availability

Scripts and data are available on a public GitHub repository: https://github.com/amob/Seasonal‐duckweed‐mbio‐effects as well as on Dryad: DOI: 10.5061/dryad.6q573n6bh.

## Appendix

**TABLE A1:** Locations of the Thompson Farm and LaRoche Pond sampling sites. Both locations are located southwest of the University of New Hampshire's campus in Durham, New Hampshire.

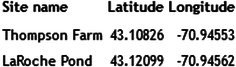

**TABLE A2:** Experimental layout including sample collection dates for Thompson Farm and LaRoche Pond.
